# Microbiota of little penguins and short-tailed shearwaters during development

**DOI:** 10.1371/journal.pone.0183117

**Published:** 2017-08-14

**Authors:** Meagan L. Dewar, John P. Y. Arnould, Theo R. Allnutt, Tamsyn Crowley, Lutz Krause, John Reynolds, Peter Dann, Stuart C. Smith

**Affiliations:** 1 School of Exercise and Nutritional Sciences, Deakin University, Burwood, Australia; 2 School of Life and Environmental Sciences, Deakin University, Burwood, Australia; 3 Bioinformatic Core Research Group, Faculty of Health, Deakin University, Waurn Ponds, Australia; 4 QIMR Berghofer Medical Research Institute, Brisbane, Australia; 5 University of Queensland Diamantina Institute, Translational Research Institute, Brisbane, Australia; 6 Deakin Biostatistics Unit, Faculty of Health, Deakin University, Burwood, Australia; 7 Biostatistics, Faculty of Medicine, Nursing and Health Sciences, Monash University, Melbourne, Australia; 8 Research Department, Phillip Island Nature Parks, Phillip Island, Australia; University of Sydney, AUSTRALIA

## Abstract

The establishment and early colonisation of the gastrointestinal (GI) tract has been recognised as a crucial stage in chick development, with pioneering microbial species responsible for influencing the development of the GI tract and influencing host health, fitness and disease status throughout life. Development of the microbiota in long lived seabirds is poorly understood. This study characterised the microbial composition of little penguin and short-tailed shearwater chicks throughout development, using Quantitative Real Time PCR (qPCR) and 16S rRNA sequencing. The results indicated that microbial development differed between the two seabird species with the short-tailed shearwater microbiota being relatively stable throughout development whilst significant fluctuations in the microbial composition and an upward trend in the abundance of Firmicutes and Bacteroidetes were observed in the little penguin. When the microbial composition of adults and chicks was compared, both species showed low similarity in microbial composition, indicating that the adult microbiota may have a negligible influence over the chick’s microbiota.

## Introduction

At birth, the gastrointestinal (GI) tract is considered to be devoid of any microbiota [1]. In mammals, the microbial communities are inherited from the mother through contact with their faecal and vaginal microbes and from breast milk [1]. In birds, however, newly hatched chicks acquire their microbiota from multiple sources including, the surface of the egg, the surrounding environment (i.e. nest) and their first meal [2–9]. In seabirds, vertical transmission of microbes via regurgitation of an undigested or partially digested meal is also said to influence microbial colonisation [10]. The GI tract is rapidly colonised by aerobic and facultative anaerobic bacteria such as *Escherichia coli* and *Streptococci* spp. immediately after birth [6, 11–14]. After initial colonisation, aerobic bacteria modify the GI tract environment, by reducing the oxygen level through oxidation/reduction. This process creates an environment that is highly favourable for facultative and obligate anaerobes [13, 15], such as *Bacteroides*, *Clostridia*, and *Bifidobacterium* [13, 16] to flourish before stabilising as an anaerobic environment that is representative of the adult GI tract.

This study examined the GI microbiome of the short-tailed shearwater (STS) (*Ardenna tenuirostris*) and the little penguin (LP) (*Eudyptula minor*). STS breed between September and April with egg laying occurring in the last week of November [17]. STS nest in burrows, lined with vegetation and lay one egg, which is incubated for a period of 50–55 days [18, 19]. In contrast to other seabirds, Procellariiformes are able to regulate body temperature much earlier, reducing their guard stage (where parents protect the chick from the cold) from 2–3 weeks to just to 2–3 days. This ability to regulate body temperature is in part due to the subcutaneous fat deposits laid down during the first few days of life [19]. Like all Procellariiformes, STS use a twofold foraging strategy during the breeding season, with adults alternating between two short foraging trips of 1–4 days, with one long foraging trip of approximately 8–19 days [20, 21]. Previous studies have documented that the diet of short-tailed shearwaters is dominated by Australian krill, *Nyctiphanes australis* [18, 21, 22]. However, in contrast to previous studies, Weimerskirch & Cherel [20], discovered that during long foraging trips, Antarctic krill and Myctophid fish also dominated the diet of STS found breeding in Australia. In Victoria, STS feed in local waters predominantly eating crustaceans, *Nyctiphanes australis* and *Paraprone clausi*, [22, 23]. Although chicks do not fledge until around 97 days old, adults begin their migration north when their chicks are around 85 days old. For the final 2–3 weeks prior to fledging chicks must survive off their endogenous fat stores [24].

At Phillip Island, Australia, LP breed between August to February [25], with peak egg laying occurring between September and October [26]. Similarly to STS, LPs nest in burrows, but will also nest under rocks, in crevices and artificial nest boxes lined with vegetation. LPs produce two eggs which are incubated for 35 days [26, 27]. Unlike STS, the guard state in LP lasts around 2–3 weeks as chicks are unable to regulate their body temperature during the early stages of development [28, 29], During the guard stage, chicks are fed on average every 1–2 days [30], whereas during the post-guard stage adults alternate between short and long foraging trips [31], with fledging occurring between 60–70 days (9–10 weeks) of age [28].

The aim of this study was to characterise the microbial community of these two seabird species during development from newly hatched chicks to fully fledged juveniles. Quantitative Real Time PCR and pyrosequencing of the 16S rRNA gene were used to identify the abundance of major taxa and microbial composition at different stages during development. To our knowledge, this is the first study to investigate the microbial composition of seabirds throughout development.

## Experimental design

### Study design and sample collection

All ethics approval was received by Phillip Island Nature Parks and Deakin University Animal Ethics Committees. Permits were received from the Department of Sustainability and Environment. Cloacal swabs were collected from little penguin (n = 9) and short-tailed shearwater chicks (n = 10) during development at the Phillip Island Nature Parks (38.4833°S, 145.2333°E). Little penguin samples were collected within the Summerland Peninsula, a previous housing estate, which had been reclaimed for penguin habitat. Due to severe compaction of the ground, burrows in the area consist of artificial wooden burrows. Burrows are lined with vegetation and excrement from previous breeding seasons. At the beginning of the breeding season, 10 nest boxes with eggs present were selected, with each nest containing two eggs. Of the 20 eggs, 16 successfully hatched, with nine chicks successfully fledging at the end of the breeding season. Short-tailed shearwater samples were collected from shearwaters nesting in natural burrows among the sand dunes of the Phillip Island Nature Parks, Penguin Parade. At the beginning of the breeding season, 20 burrows with eggs were randomly selected. Of the 20 eggs, 17 successfully hatched, with 10 chicks successfully fledging at the end of the breeding season.

Following hatching, cloacal swabs were collected from each chick directly from the cloacae using Eswabs (Copan, Italy). On the same day, each week (LPs)/fortnight (STS), chicks would be removed from their nest, weighed, inked (red or green food dye) for identification purposes (little penguin chicks), swabbed and then placed back into the nest within 5–10 minutes to avoid stress. Both male and female adults were captured at night when returning to their nests. Due to their feeding strategies, adult shearwaters and little penguin adults were not always present in the colony at times of sampling. Samples were collected via direct cloacal swab. Samples were placed into an amine solution for preservation of the DNA and stored in liquid nitrogen (-196°C) for shipment and then stored at -80°C until DNA extraction.

### DNA extraction and real time PCR

DNA was extracted from the swabs using the Qiagen^™^ QIAamp DNA Stool Mini Kit (Hilden, Germany) following the manufacturer’s instructions. The major phyla selected for analysis in this study were selected on the basis of previous studies that had examined the predominant gastrointestinal microbiota of vertebrates [22–27], which included Firmicutes, Bacteroidetes, Actinobacteria and Proteobacteria [28]. The primer sequences and annealing temperatures for the chosen bacterial groups can be found in Dewar et al [32]. The quantitative real time PCR was performed on a Stratagene MX3000P. Each PCR reaction mixture comprised of 5 μl of Brilliant II SYBR green (Stratagene^™^), 20 pmol/μL of forward and reverse primer, 2 ng of template DNA and made up to a final volume of 20 μl with nuclease free water. The cycling conditions were 95°C for 2 mins, followed by 40 cycles of 95°C for 5 secs, followed by annealing temperature [32] for 30 secs with all samples run in triplicate. Bacterial concentration was determined by comparing the threshold value (Ct. Values) with a standard curve. The standard curve was created by using a serial 10-fold dilution from DNA extracted from a pure culture of *E*. *coli* ranging from 10^2^–10^10^ CFU/g.

### PCR amplification and 16S rRNA pyrosequencing

From the original chick samples, one sample per fortnight from four Individuals for each species was amplified using universal primers Roche adapter A (5’GCC TCC CTC GCG CCA TCA GT-3’) and reverse 338R (5’-CAT GCT GCC TCC CGT AGG AGT-3’) to amplify the V2-V3 region as per Dewar et al [32]. Due to irregular attendance by adults during the breeding season, samples during the guard stage (1^st^ week post hatching) were used for analysis. 16S rDNA from faecal samples from four individuals per species, was amplified as above. Following amplification samples were pooled with the attachment of MID tag barcodes to ensure that bacterial DNA from all members of each species was equally represented (i.e. Barcode 338R_BC0500 “CAGCTCAACTA” was attached to all week 1 samples). Samples were then sequenced on the Roche/454 FLX Genome Sequencer by Engencore (USA) according to Fierer et al [33].

### Data processing and analysis

Unprocessed reads were submitted to the NCBI SRA archive (Bioproject PRJNA347517). The UPARSE 454 pipeline was used for 16S amplicon analysis [34] with a 3% cluster radius. Reads were filtered, prior to UPARSE, to a minimum length of 300 bp. The RDP 16S database v16 (http://drive5.com/utax/data/rdp_v16.tar.gz) was used to taxonomically classify OTUs in UPARSE with a 90% confidence threshold. UPARSE detected 498 OTUs. OTUs with abundance less than 0.1% of the total number of reads or occurrence in less than two samples across all samples were removed from analysis, 62 OTUs remained. QIIME 1.8 [35] was used to calculate diversity metrics and statistically compare diversity and OTU abundance between species. OTU abundance was CSS normalised prior to statistical analysis [36]. Pearson correlations were calculated to examine changes in OTU abundance and diversity over time. All commands and associated python scripts in the analysis are provided in the GitHub repository: https://github.com/bioinformatics-deakin/Dewar_and_Allnutt_et.al_2016. Principal coordinates analysis (PCoA) was performed on weighted UniFrac distances [37] calculated from a maximum likelihood tree (FastTree v2.3.1, [38]) of the filtered OTUs. Beta diversity was analysed by AMOVA [39].

### Prediction of the functions of the microbial communities

PICRUSt v1.0 [40] was used to predict functional pathways of the GI communities from the 16S rDNA data using the KEGG pathway database. Differences in abundance of pathways were tested using ANOVA in QIIME. All scripts and commands used in bioinformatic analyses are detailed in: https://github.com/bioinformatics-deakin/Dewar_and_Allnutt_et.al_2016.

### qPCR statistical analysis

In order to explore the variation in bacterial counts from the qPCR analysis, linear mixed models with individual birds and samples from birds, as random effects and the common sampling times (weeks) as a fixed effect were fitted to the log (base 10) transformed bacterial counts–this variance-stabilizing transformation was deemed to be necessary after inspection of diagnostic plots of Studentized residuals versus predicted values [41] for analyses based on the raw counts.

Six types of linear mixed models were investigated (three autocorrelation models for the repeated measurements by two models for the effect of time):

Repeated Measurements:

1. No autocorrelation in the repeated counts from the penguin chicks and homogeneous variances at each assessment (scaled identity model).
2. No autocorrelation in the repeated counts from the penguin chicks and heterogeneous variances at each assessment (diagonal model).
3. First order autoregressive process for the co-variation in the repeated counts (AR (1) model) and homogeneous variances.

Time effect:

1. Week as a categorical factor (i.e. no systematic trend)
2. Week as a linear trend.

For each abundance measure (i.e. log-transformed count), the fitted model with the smallest value of the Akaike Information Criterion (AIC) [42] was selected and the significance of the association of the common assessment times with variation in abundance was assessed using the F-test. If the selected model included week as a categorical factor, weekly means and their standard errors, as calculated from the fitted fixed model, were reported otherwise, if the selected model included week as a linear trend, the estimated intercept and slope coefficients, and their standard errors, were reported. All analyses used the mixed model procedures in the IBM SPSS Statistics package (Version 20) and unless otherwise stated, all abundance measures are reported on the log (base 10) scale.

## Results

### Quantitative real time PCR

**Fig 1:**
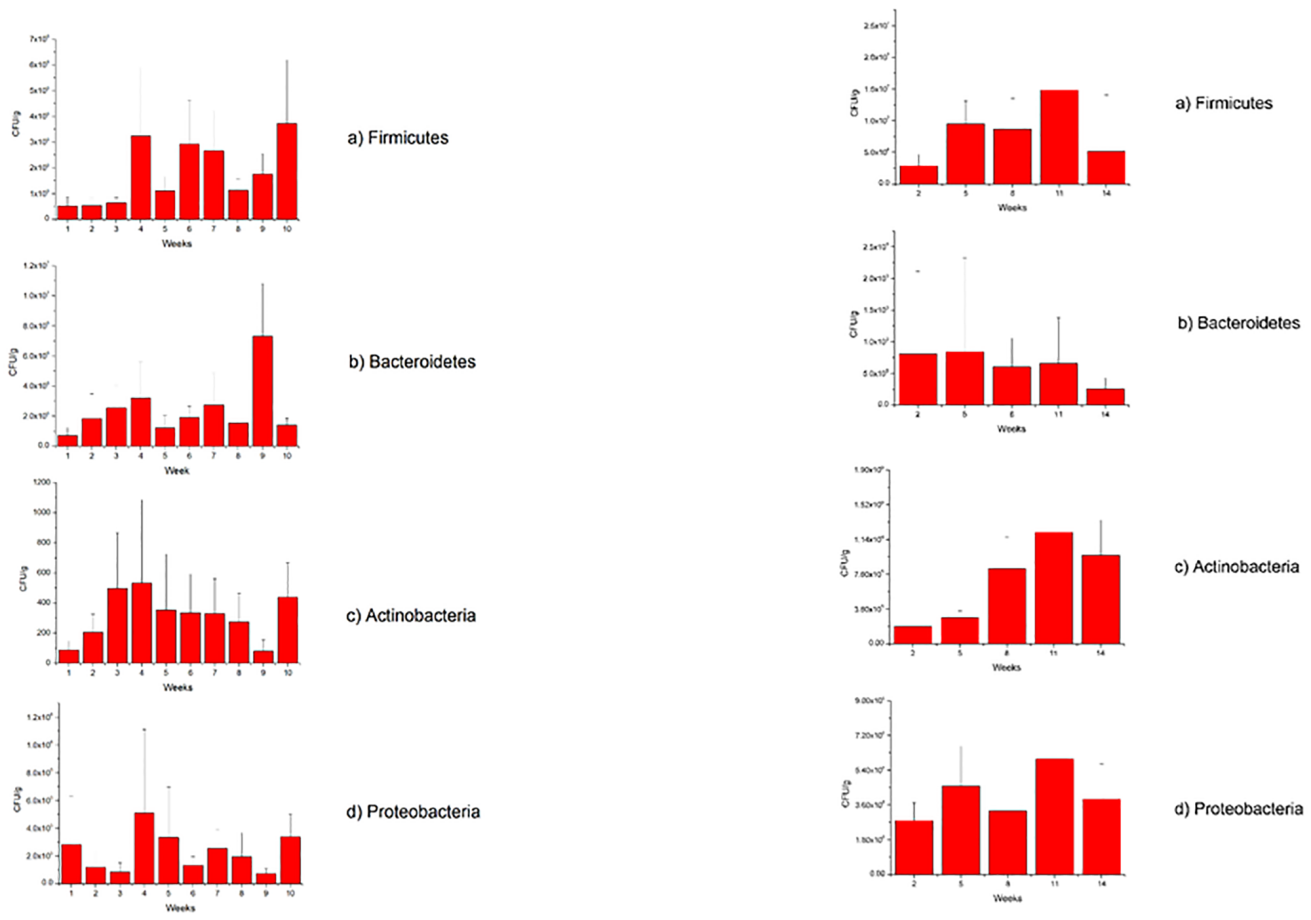
a. Mean abundance of the major phyla; Actinobacteria (a), Bacteroidetes (b), Firmicutes (c) and Proteobacteria (d) during development in little penguin chicks b. Mean abundance of the major phyla; Actinobacteria (a), Bacteroidetes (b), Firmicutes (c) and Proteobacteria (d) during development in short-tailed shearwater chicks.

The abundance of four major phyla; Firmicutes, Bacteroidetes, Actinobacteria and Proteobacteria was analysed using quantitative real time PCR. In LPs, Bacteroidetes and Firmicutes appeared to dominate the microbial composition throughout development, followed by Proteobacteria. Actinobacteria had the lowest abundance. During weeks 1–3, 5, 7–9 Firmicutes appeared to dominate the microbial composition, whilst during weeks 4, 6 and 10 *Bacteroidetes* were prevalent (Fig 1a). When analysing the data using a linear regression model with time as a covariate, a significant increase was observed for both Firmicutes and *Bacteroidetes* from hatching to fledging. For STS chicks, Firmicutes appeared to dominate the microbial composition throughout the entire breeding season. When analysing the data using a linear regression model with time as a covariate, no significant trends in the abundance of the major phyla were detected in the STS chicks (Fig 1b).

When comparing the microbial composition of the two seabird species during development, significant species effects for Actinobacteria (P <0.001), Firmicutes (P <0.005) and Proteobacteria (P <0.001) were observed with LP log(Actino) values being, on average, -3.602 (± 0.384) below STS values, LP log(Firm) values being, on average, -0.686 (± 0.277) below STS values and LP log(Proteo) values being, on average, -1.154 (± 0.206) below STS values. No significant species effect for Bacteroidetes (P = 0.485) was noted with LP log(Bacter) values being, on average, 0.193 (± 0.248) above STS value. With time fitted as a continuous covariate there was a significant linear trend in Actinobacteria (P <0.005) common to both species with log(Actino) values increasing 0.0585 (±0.0194) per week. However, the species by time interaction was not significant (P = 0.207). A significant trend, common to both species, for time fitted as a continuous covariate was also significant for Firmicutes (P<0.001), with log(Firm) values increasing 0.0360 (±0.0211) per week. The species by time interaction was significant (P = 0.033) with an additional linear increase of 0.0729 (±0.0338) per week in the LP species. Whilst for Bacteroidetes (P = 0.557) and Proteobacteria (P = 0.302) there was no linear trend for time. However, the species by time interaction was significant for Bacteroidetes (P <0.001) with a linear increase of 0.0987 (±0.0217) per week in the LP species.

### PCoA and beta diversity

**Fig 2:**
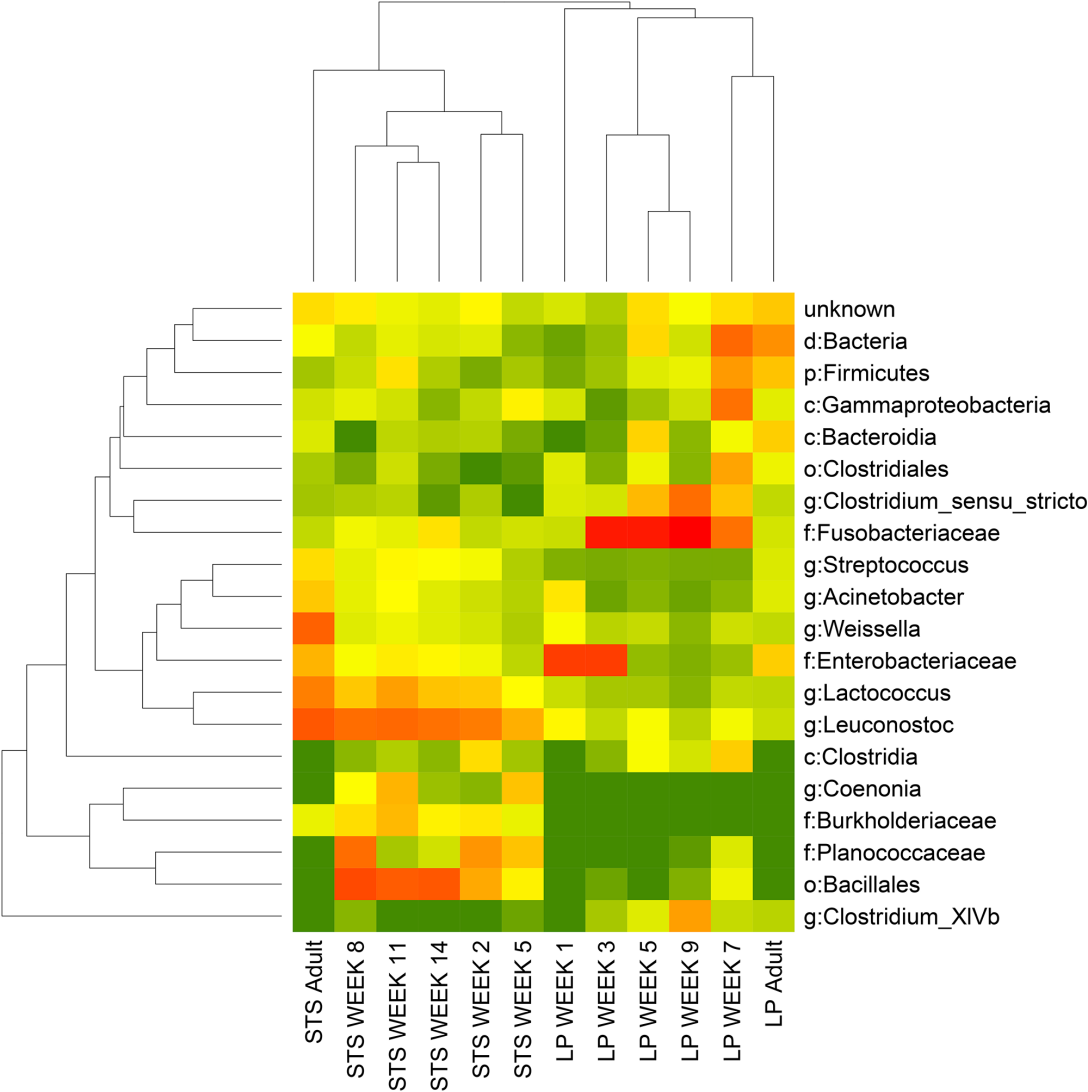
Heat map of 20 most abundant OTUs in STS and LP chicks and adults. Assigned taxonomy (UPARSE, 90% confidence) is shown for each OTU.

UPARSE identified 498 OTUs at the 97% identify level. After filtering to 0.1% abundance and occurrence in at least two samples, 63 OTUs remained. Fig 2 shows a heat map of OTU abundance for the 20 most abundant OTUs. STS and LP species form distinct clusters with significantly different abundances of taxa: at the class level, Flavobacteria, Betaproteobacteria, and Bacilli were more abundant in STS than LP (FDR = 0.002, 0.004, and 0.002 respectively); and Clostridia and Fusobacteria were more common in LP than STS (FDR = 0.01, 0.05 respectively). At the order level, Flavobacteriales and Burkholderiales were more abundant in STS than LP (FDR = 0.005, 1.24 x 10^−11^ respectively); and Clostridiales and Fusobacteriales were more common in LP than STS (FDR = 0.01, 0.02 respectively). At the family level, Corynebacteriaceae, Veillonellaceae, Flavobacteriaceae, Burkholderiaceae, Planococcaceae, Streptococcaceae, Leuconostocaceae were more abundant in STS than LP (FDR = 0.03, 0.03, 0.0003, 9.4242 x10^-10^, 0.007, 0.0001, 0.0003 respectively); and Eubacteriaceae and Fusobacteriaceae were more common in LP than STS (FDR = 0.07, 0.03 respectively). At the genus level, *Chryseobacterium*, *Burkholderia*, *Streptococcus*, *Acinetobacter*, *Coenonia*, *Lactococcus*, and *Leuconostoc* were more abundant in STS than LP (FDR = 0.002, 1.5 x 10^−5^, 0.003, 0.05, 0.006, 7.8 x 10^−6^, 0.0001 respectively); *Psychrobacter* and *Clostridium_XlVb* were more common in LP than STS (FDR = 0.05, 0.04 respectively).

**Fig 3:**
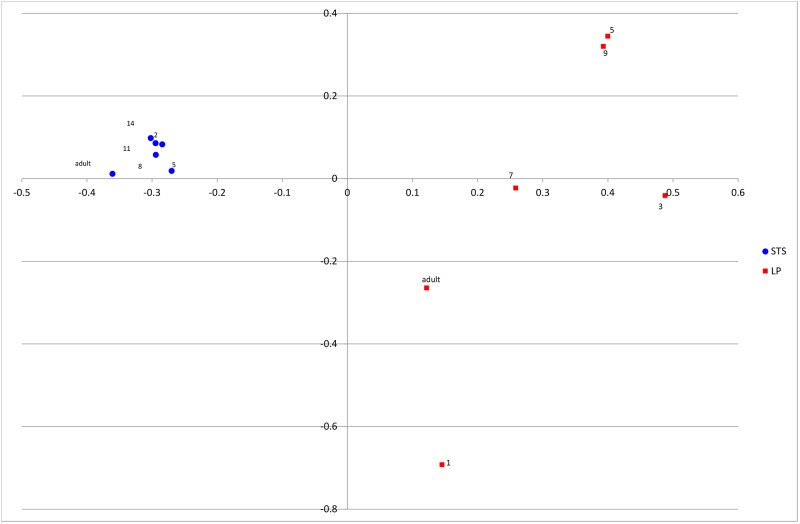
PCoA of weighted UniFrac distances among STS and LP samples. Points are labelled with the chick age (weeks). LP = little penguin, STS = short tailed shearwater. PCO 1 and 2 shown, described 49% and 23% of total variation respectively.

Fig 3 shows a PCoA of UniFrac distances of all STS and LP samples. Microbial samples clearly cluster by host species, indicating that STS and LP have very different GI bacterial populations. STS also forms a tighter cluster than LP, indicating a lower diversity of OTUs. There is no evidence of clustering by age. AMOVA identified a significant proportion of variation in UniFrac distance between species, 43%, P = 0.001.

### Alpha diversity

Alpha diversity measures, Shannon's, Simpson's and observed OTUs, calculated for STS and LP species, are shown in Table 1. Rarefaction depth was set to the minimum for the filtered OTU set (2104). The number of observed OTUs was significantly different between species (LP mean = 25; STS mean = 32; P = 0.025). Shannon's and Simpson's diversity measures were not significantly different between species. Increase in alpha diversity was not evident during the development period studied, with no significant correlation for all measures (Table 2).

**Table 1:** Alpha diversity measures based on a rarefaction depth of 2104.

Measure	LP mean	LP Std Dev	STS mean	STS Std Dev	t stat	p-value
**Observed otus**	25.05	4.998	32.83	4.978	-2.467	0.025
**Shannon's**	2.241	1.017	3.086	0.389	-1.737	0.103
**Simpson**	0.597	0.211	0.793	0.058	-1.995	0.077

Statistical comparison was the non-parametric t-test implemented by QIIME v 1.8 with 1000 bootstrap replicates. LP = little penguin, STS = short tailed shearwater

**Table 2:** Alpha diversity correlated with development age of chicks.

Age (weeks)														
Measure	STS					LP					STS R	STS p-value	LP R	LP p-value
	2	5	8	11	14	1	3	5	7	9				
Obs. OTUs	33	39	38	39	36	29	35	39	39	34	0.372	0.269	0.534	0.177
Shannon	4.97	5.2	5.15	5.21	5.08	4.76	5.03	5.18	5.16	4.96	0.367	0.272	0.480	0.207
Simpson	0.97	0.97	0.97	0.97	0.97	0.96	0.97	0.97	0.97	0.96	0.363	0.274	0.418	0.242

### Taxonomic composition

**Fig 4:**
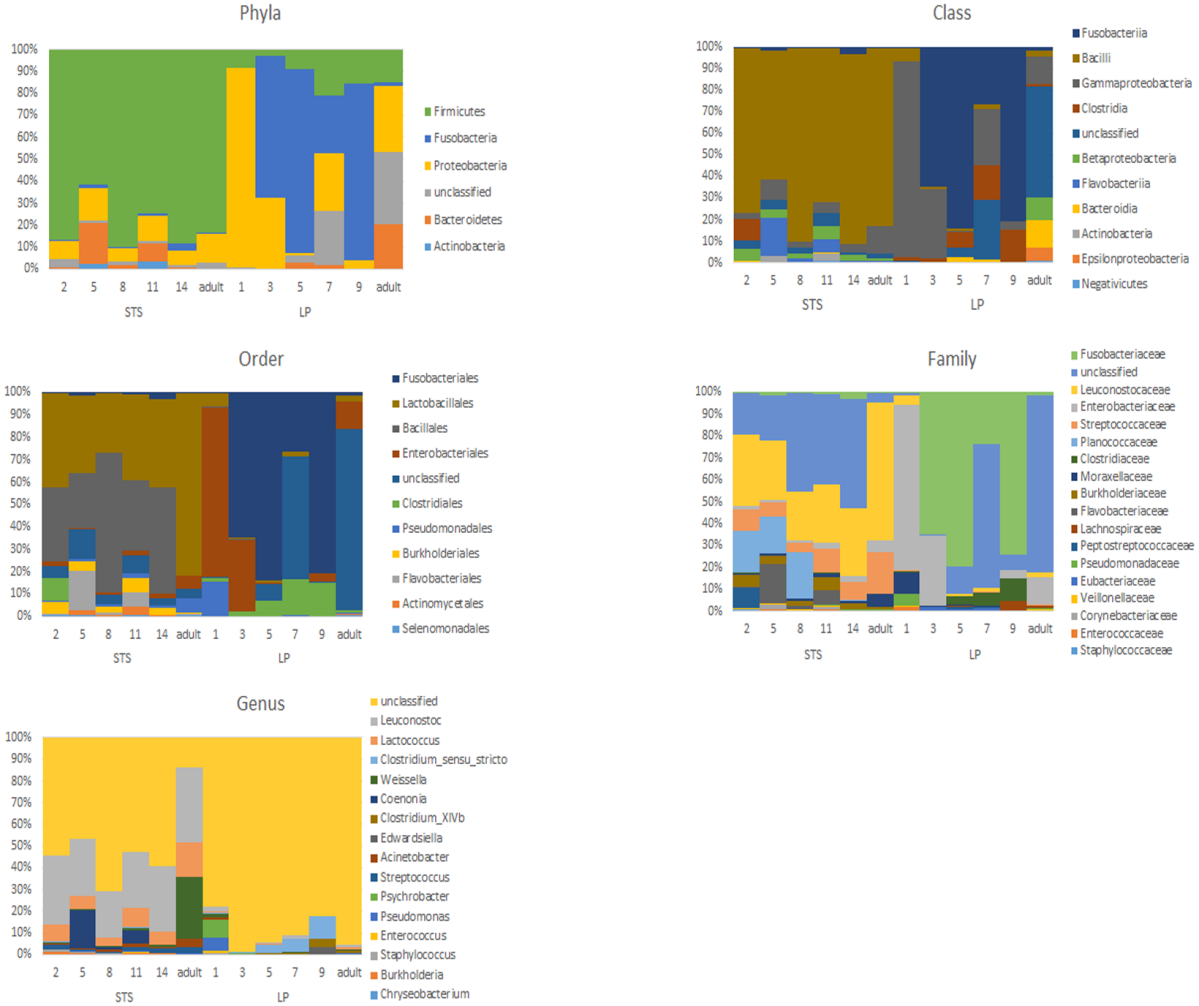
Bar charts showing the taxonomic diversity of LP and STS samples (proportion of total reads assigned to each taxon). Chick age is shown in weeks.

Bar charts of taxonomic composition are shown in Fig 4. The distribution of major phyla varied between STS and LP. Firmicutes were significantly more abundant in STS than LP (ANOVA, P = 0.023). Fusobacteria appeared more common in LP, but were not statistically significant—reflecting the large variability in this Phylum in LP, where it was absent from the youngest LP chick sample and the LP adult. At the class level, only Bacilli were significant, being more common in STS (P = 0.007). At the level of order, Lactobacillales, Burkholderiales, and Selenomonadales were more prevalent in STS than LP (P = 0.044, 0.044 and 0.049 respectively). There were no significant differences at family level. At genus level five taxa were significantly more abundant in STS than LP: *Leuconostoc*, *Chryseobacterium*, *Streptococcus*, *Burholderia*, and *Lactococus*; P = 0.004, 0.014, 0.033, and 0.042 respectively. Taxon abundance was not correlated (Pearson correlation, t-test P > 0.05) to chick age for any taxa.

### Inferred functional diversity

PICRUSt identified 219 biological processes in both little penguins and short-tailed shearwaters including pathways in cellular processes, Environmental Information Processing, Genetic information processing and metabolism (S1 Table). None of the identified processes differed significantly between species and none correlated significantly with chick age.

## Discussion

Establishment and early colonisation of the GI tract has been recognised as a crucial stage in chick development with pioneering microbial species responsible for influencing the GI tract pH, oxygen levels, mucosal structure and immunity, and therefore, these pioneering species influence a host’s overall health and disease status throughout life [43–45]. Immediately after birth/hatching, the GI microbiota is rapidly colonised and undergoes successional changes until a dense and stable community is achieved. In most vertebrates, this process can take anywhere from 40 days [6, 12, 46–48] to 2 years [13, 49–51]. In black legged kittiwakes (*Rissa tridactyla*) chicks are colonised by many transient species, gradually transitioning into a more stable microbiota that is representative of the adult microbiota [52]. To date there are limited data available on the GI microbiota of long lived seabirds, and the microbial succession that occurs during development. Accordingly, this study aimed to examine the establishment and microbial succession that occurs in little penguin and short-tailed shearwater chicks throughout development.

### Little penguins

As with other young vertebrates, the little penguin microbiota is dominated by aerobic and facultative anaerobic bacteria, with the total population, estimated at over 10^7^ CFU/ml by 7 days, with a significant upward trend observed throughout development in Firmicutes and Bacteroidetes populations [134,166]. During the early stages of development, the microbiota is dominated by members of the family Enterobacteriaceae which have been identified as pioneering populations associated with gut maturation and development, absorption of dietary fats and lipids, and soluble vitamins, enhance the immune system, alteration of lipoprotein profiles, modulation of proinflammatory and anti-inflammatory gene expression. However, these microbes have also been implicated in GI pathologies, brain-gut axis, and neurological conditions [53–60]. While *Psychrobacter*, isn’t a normal component of the infant microbiota, it has been shown to have probiotic properties boosting the immune system and weight gain of Groupers [61]. These pioneering microbes are also said to alter the GI environment in favour of the growth of anaerobic bacteria. Similar to other studies, the microbiota of the little penguin began to transition from an aerobic/facultative anaerobic microbial population, to a strict anaerobic population by week 3, and continued throughout development, with the anaerobic population being dominated by butyrate producing microbes by week 5. In poultry, butyrate has been shown to not only boost the immune system and protect against colonisation by pathogenic bacteria, but has also been shown to influence host adiposity [62]. In little penguins, there is a strong link between host body mass and fledging survival. Therefore, the high abundance of butyrate producing microbes and presence of *Psychrobacter* in little penguin chicks could influence chick body mass positively and therefore fledging survival. However, this would require further analysis.

### Short-tailed shearwater

Throughout the development of short-tailed shearwater chicks, the microbiota is dominated by members of the phylum Firmicutes. In humans and mice, the ratio of Firmicutes to Bacteroidetes has been shown to influence a host’s adiposity level by enhancing energy extraction and modulating genes that regulate fat storage [63–67] and is currently considered to play a crucial role in host adiposity and obesity. In obese individuals, the ratio of Firmicutes to Bacteroidetes is 100:1, whereas in their lean counterparts the ratio is 10:1 [63, 64, 67]. Similarly to adult short-tailed shearwaters, the ratio of Firmicutes to Bacteroidetes in short-tailed shearwater chicks ranged from 73:1 to 85:1 and are similar to that of obese humans, mice and Australian fur seals, except for week 5, where the ratio was 62:18 [63, 64, 67, 68]. Following hatching, the body mass of short-tailed shearwater chicks rapidly increased and by week 7, the body mass of chicks exceeded that of the adult shearwater by 15–50% [69]. While many factors, such as a high lipid diet and gut physiology, may influence body mass, the high abundance of Firmicutes of the short-tailed shearwater chick may confer a predisposition towards developing body fat stores. A chick’s ability to rapidly accumulate large fat reserves enables chicks to survive the long periods between feeds and is essential for survival post fledging [70]. Unlike penguins, the microbiota of short-tailed shearwaters showed little variation in composition and diversity throughout development; even during the final stages of development when adults abandon their chicks to return to the northern hemisphere, leaving chicks to survive off their endogenous fat reserves [24]. However due to the absence of data available on the role of microbes in seabirds, the importance of the microbiota for the survival of little penguins and short-tailed shearwaters requires more study.

### Species differences

Establishment and early colonisation of the GI tract has been recognised as a crucial stage in infant development with pioneering species responsible for influencing the GI tract pH, oxygen levels, mucosal structure and immunity [43–45]. Immediately after hatching/birth, the GI microbiota is rapidly colonised and undergoes successional changes until a dense and stable community is achieved. In most vertebrates, this process can take anywhere from 40 days [6, 12, 46–48] to 2 years [13, 49–51]. The results from this study identified that the development of the seabird microbiota differs greatly between species. In the little penguin, there were significant differences throughout development and significant upward trends in the abundance of Firmicutes and Bacteroidetes, whereas, in the short-tailed shearwater there were no significant differences in the microbial composition throughout development and appeared to be relatively stable throughout development with a high level of similarity between each age class.

The microbial composition of the little penguin is dominated by microbes that are responsible for the absorption of dietary fats, lipids, and soluble vitamins, they maintain intestinal barrier function, regulate triglycerides, impact intestinal permeability, activate intestine-brain-liver neural axis, enhance the immune system, alter lipoprotein profiles, protect against stress-induced lesions, modulate proinflammatory and anti-inflammatory gene expression, butyrate production [71] and strengthen epithelial cell barrier properties. However, these microbes have also been implicated in GI pathologies, brain-gut axis, and a few neurological conditions [53–59]. In the short-tailed shearwater chick, however, the GI microbiota appears to be associated with soil and lactic acid bacteria that are not commonly associated with the GI tract.

In seabirds, pre-fledging body mass is associated with juvenile survival and therefore, the accumulation of fat reserves is important for post fledging survival. In little penguin chicks the relatively high abundance of butyrate-producing microbes could influence body mass, as previously observed in poultry [62]. Although the ratio of Firmicutes to Bacteroidetes strongly resembles that of obese humans, mice and Australian pinnipeds, the microbes within the phylum Firmicutes (*Lysinibacillus*, *Leuconostoc* and *Lactococcus*) have previously not been associated with energy extraction, lipid accumulation or obesity.

The differences between little penguins and short-tailed shearwaters could be in part due to differences in diet composition, digestive physiology, nesting environment and host phylogeny, all of which have been previously shown to strongly influence the microbial composition of vertebrates [7, 72–74]. According to the PICRUSt predictions, the microbiome may be involved in a number of metabolic, cellular, environmental and genetic information processing. No significant differences between functional pathways and species or age could be found. It must be noted that PICRUSt is a computation model that predicts the functional composition of the microbiome, by using marker gene data and a database of reference genomes [40], and are therefore a prediction of the potential functional role of microbes in seabird chicks. Therefore, further metagenomic and functional studies are required to identify the role these specific microbes play in seabird chicks.

## Conclusion

The results from this study identified that the development of the seabird microbiota differs greatly between LP and STS. In the little penguin, there were significant differences in changes throughout development, with a significant upward trend in the abundance of Firmicutes and Bacteroidetes. Whereas, in the short-tailed shearwater there were no significant differences throughout development with a high level of similarity between each age class. However, there is low similarity in the microbial composition between adults and chicks for both the STS and LP, indicating that the adult’s microbiota may have a negligible influence over the chick’s microbiota. The differences between little penguins and short-tailed shearwaters could be in part due to differences in diet composition, digestive physiology, nesting environment, host phylogeny and reproductive strategy, all of which have been previously shown to strongly influence the microbial composition of vertebrates [7, 72–74].

In seabirds, fledging body mass is associated with juvenile survival [75–78] and therefore, the accumulation of fat reserves is important for post fledging survival. In little penguin chicks the relatively high abundance of butyrate-producing microbes could influence body mass, as previously observed in poultry [62]. Although the ratio of Firmicutes to Bacteroidetes in short-tailed shearwaters strongly resembles that of obese humans, mice and Australian Sea Lions (*Neophoca cinerea*), the microbes within the phylum Firmicutes (*Lysinibacillus*, *Leuconostoc* and *Lactococcus*) have not previously been associated with energy extraction, lipid accumulation or obesity. Due to the absence of information on the functional role of the dominant microbiota, we are unable to elucidate the role of the GI microbiota in fledging survival of little penguins and short-tailed shearwaters.

## Supporting information

S1 Table Functional pathways predicted by PICRUSt and statistical analysis. (XLSX)
